# Impact of sustentaculum tali screw positioning on radiographic and functional outcomes in calcaneal fractures

**DOI:** 10.1186/s13018-023-04521-x

**Published:** 2024-02-12

**Authors:** Zihua Li, Fajiao Xiao, Hui Huang, Jiang Xia, Haichao Zhou, Bing Li, Yunfeng Yang

**Affiliations:** 1 Department of Orthopedics, Shanghai Tongji Hospital, School of Medicine, Tongji University, Shanghai, 200065 China; 2 Department of Orthopedics, Shanghai Tenth People’s Hospital, School of Medicine, Tongji University, Shanghai, 200072 China

**Keywords:** Calcaneal fracture, Functional outcome, Open reduction and internal fixation, Sustentaculum tali

## Abstract

**Background:**

To investigate whether accurate placement of sustentaculum tali screws have the impacts on the clinical efficacy of calcaneal fractures.

**Methods:**

A retrospective analysis of 72 cases (73 feet) of calcaneal fractures from September 2015 to September 2019 treated with open reduction and internal fixation with sustentaculum tali screws was conducted. Patients were divided into the sustentaculum tali fixation group (ST group) and the sustentaculum fragment fixation group (STF group) according to the location of the sustentaculum tali screw placement. The functional outcomes at preoperative, 7 days and 1 year postoperative were collected and analyzed.

**Results:**

In the ST group (40 feet), the Gissane's angle altered from (109.89 ± 12.13)° to (121.23 ± 9.34)° and (119.08 ± 8.31)° at 7 days and 1 year postoperative, respectively. For Böhler’s angles altered from (11.44 ± 5.94)°, to (31.39 ± 7.54)°, and (30.61 ± 7.94)° at 7 days and 1 year postoperative, respectively. In the STF group (33 feet), Gissane’s angle altered from (110.47 ± 14.45)°, to (122.08 ± 8.84)°, and (120.67 ± 9.07)° and Böhler’s angle altered from (11.32 ± 6.77)°, to (28.82 ± 8.52)°, and (28.25 ± 9.13)° (*P* < 0.001). However, there was no statistically significant difference in functional outcomes at 1 week after surgery and 1 year after surgery (*P* > 0.05). The AOFAS scores at the final follow-up of the two groups: ST group (88.95 ± 6.16) and STF group (89.78 ± 8.76); VAS scores, ST group (0.83 ± 0.98) and STF group (1.03 ± 1.59), all differences were not statistically significant (*P* > 0.05).

**Conclusion:**

The position of sustentaculum tali screws has no significant difference on the short-term clinical outcome in patients with calcaneal fractures, while reliable fixation of screws to sustentaculum tali fragment can achieve similar clinical outcome.

*Level of evidence V*.

## Introduction

Calcaneal fractures, accounting for 2% of all bodily fractures, predominantly affect the posterior articular surface, with over 60% of these injuries occurring there [1], 2 Sanders’ classification is typically used for treatment and prognosis, focusing on this surface [3], 4. The posterior articular surface extends medially as the sustentaculum tali, important for weight-bearing and rarely displaced in fractures [5], 6. The sustentaculum tali's surrounding soft tissues and medial calcaneal cortex, exhibiting minimal displacement in calcaneal fractures, form a dense trabecular, medial weight-bearing column that integrates with the posterior sub-articular surface trabecula, providing a key anchor point for fracture repositioning and fixation. Ideal surgical treatment for calcaneal fractures typically involves screw fixation to the sustentaculum tali through the fracture block under the calcaneus's posterior articular surface, but achieving precise screw placement is challenging, with experienced surgeons attaining only 60–80% accuracy rates [7], 8. In addition, repeated intraoperative adjustments of screws in calcaneal fracture surgeries can cause additional injury to patients, raising questions about the necessity of precise screw placement in the sustentaculum tali. Studies show inconsistent surgical outcomes between groups with sustentaculum tali fixation and those without, highlighting a lack of clinical data linking precise screw placement to the final outcome. Moreover, imaging data shows that the medial fragment around the sustentaculum tali in calcaneal fractures often involves a larger area than simple sustentaculum tali fractures. This suggests the need to investigate whether screw fixation to the medial fragment could yield similar clinical outcomes as precise sustentaculum tali fixation. Consequently, a new approach, sustentaculum fragment fixation, was proposed and tested by comparing clinical outcomes of precise sustentaculum tali screw placement versus medial fragment screw placement in calcaneal fracture treatments from September 2015 to September 2019.

## Methods

### General information

We conducted a retrospective review of 72 patients with 73 feet (40 in the ST group and 33 in the STF group) who had been diagnosed as calcaneal fracture and had undergone ORIF surgery from September 2015 to September 2019. Basic demographic and clinical characteristic of the patients were collected including age, gender, type of fracture, incision approach, type of internal fixation, functional outcomes, radiological images, and follow-up time. This study was ethically approved by the Ethics Board of our hospital, reference number K-2022-003, in accordance with the Declaration of Helsinki Ethical Principles for Medical Research Involving Human Subjects. The report of this study adheres to the statement guideline: ‘Strengthening the Reporting of Observational Studies in Epidemiology’ (STROBE).

**Fig. 1:**
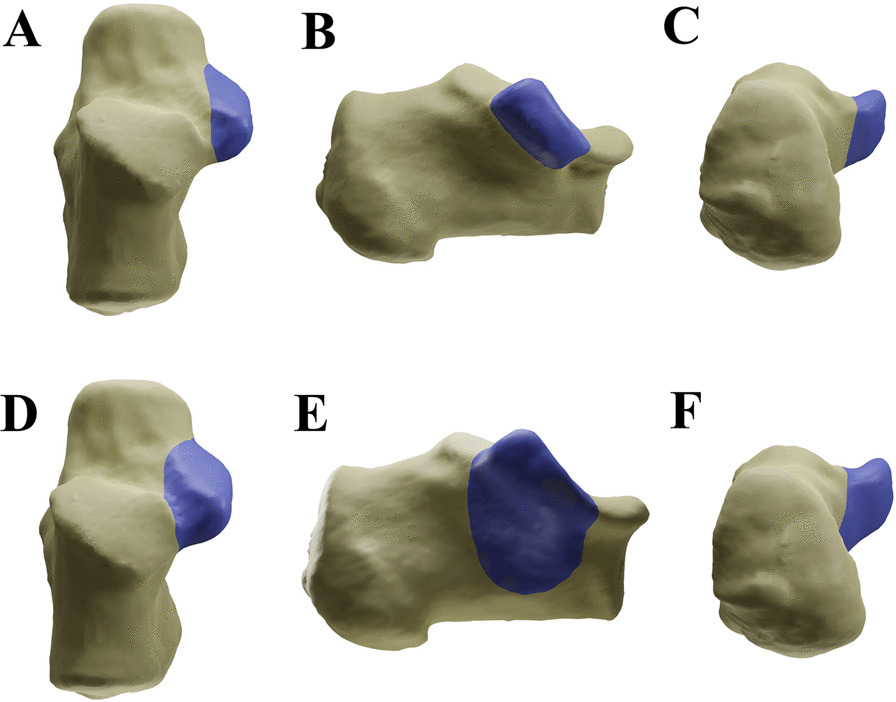
Illustration of the range of ST and STF. **A** The vertical view of ST. **B** The medial view of ST. **C** The axial view of ST. **D** The vertical view of STF. **E** The medial view of STF. **F** The axial view of STF. *ST* Sustentaculum Tali; *STF* Sustentaculum Tali Fragment

**Fig. 2:**
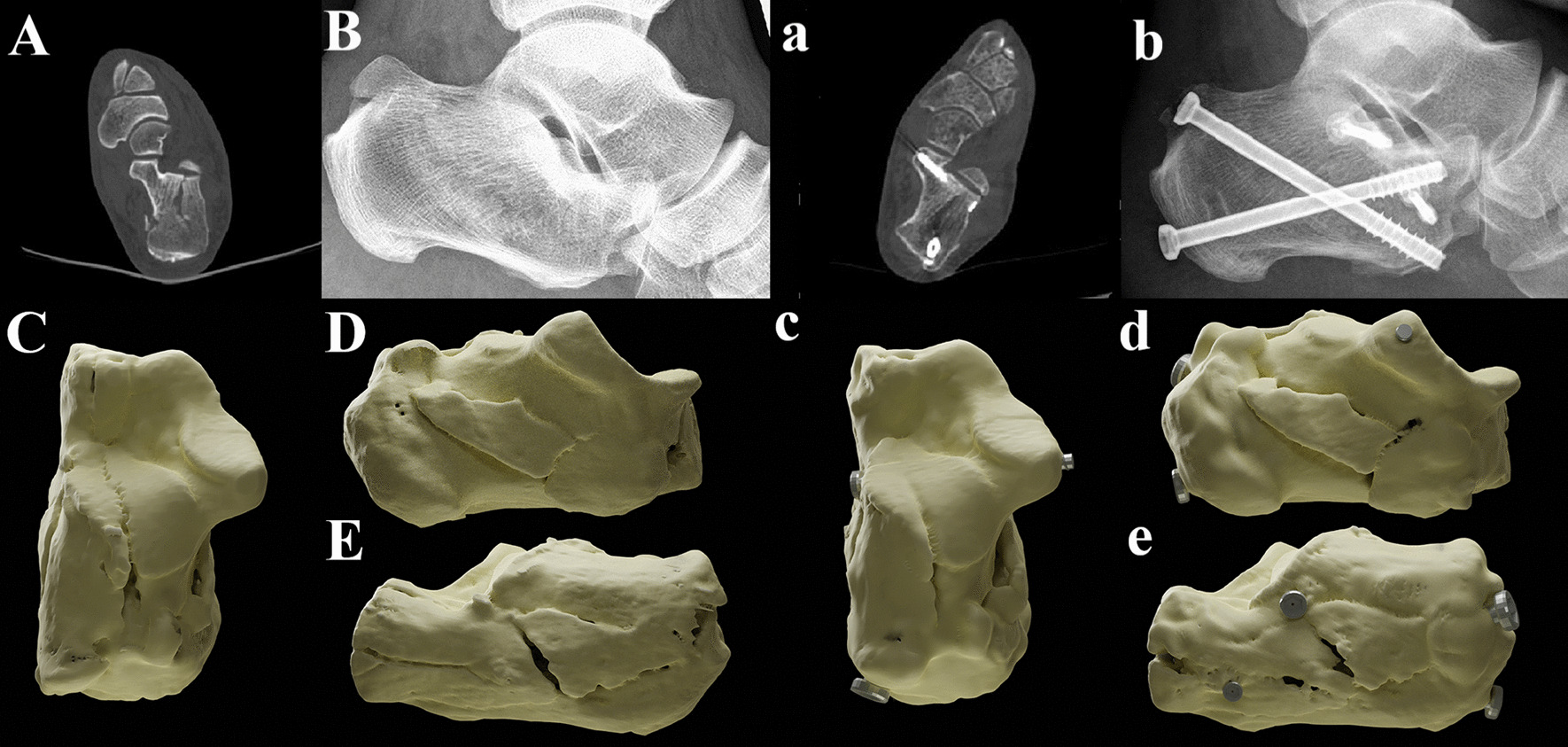
A typical case of ST group. **A**, **B** The preoperative CT scan and X-ray of the patient. **C** The vertical view of three-dimensional (3D) reconstructed illustrator before surgery. (D) The medial view of 3D illustrator before surgery. **E** The lateral view. **a**, **b** The postoperative CT scan and X-ray of the patient. **c** The vertical view of 3D reconstructed illustrator before surgery. **d** The medial view of 3D illustrator before surgery

**Fig. 3:**
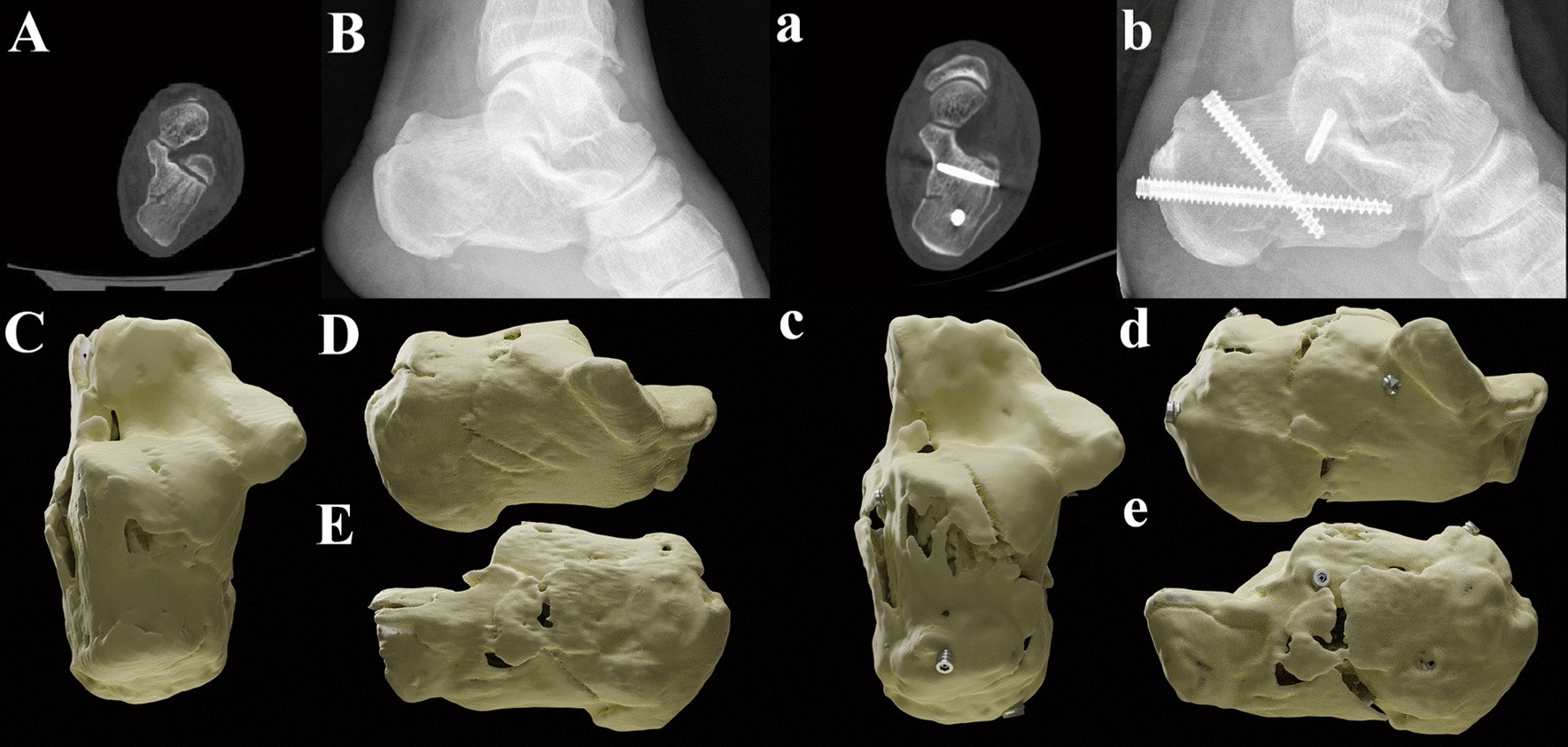
A typical case of STF group. **A**, **B** The preoperative CT scan and X-ray of the patient. **C** The vertical view of three-dimensional (3D) reconstructed illustrator before surgery. **D** The medial view of 3D illustrator before surgery. **E** The lateral view. **a**, **b** The postoperative CT scan and X-ray of the patient. **c** The vertical view of 3D reconstructed illustrator before surgery. **d** The medial view of 3D illustrator before surgery

Postoperative CT imaging data Dicom files were collected in all cases, and a three-dimensional model was created with Mimics 20.0 and engraved with Blender 3.1.2, based on the anatomical measurements of Jiong Mei et al. as the specific localization of the sustentaculum tali [9–11]. Patients will be divided into ST group if sustentaculum tali screw penetrated medial wall point located within the range of the sustentaculum tali; While STF group (Figs. 1, 2, 3) represents patients with sustentaculum tali screw penetration points not within the range of the sustentaculum tali but within the sustentaculum fragment: The CT images of the calcaneus were examined layer by layer in the cross-sectional and coronal planes, and the larger medial fragment, including the sustentaculum tali, was the cortical bone migration area of the sustentaculum tail, bounded by the first continuous bone trabecula on the lateral side of the central triangle of the calcaneus. The study included cases with fracture of the sustentaculum fragment that were classified into the ST group or the STF group, which were fixed with a sustentaculum tail screw placed through the lateral wall of the calcaneus as well (at least one screw was fixed to the sustentaculum tail or the sustentaculum fragment in each case).

### Inclusion and exclusion criteria

Patients were enrolled if they met the following eligibility criteria: (1) Fractures classified as Sanders type II-IV, (2) closed and fresh fractures with pain, swollen soft tissue and limited range of motion, (3) follow-up period at least 1 year. The exclusion criteria were as follows: (1) fractures classified as Sanders I; (2) sustentaculum tali fracture or malunion, nonunion, open fractures; (3) associated or multiple trauma; and (4) severe systemic diseases and commodity.

### Preoperative treatment and surgical technique

Generally, patients were given symptomatic treatment to reduce swelling and pain, and open reduction and internal fixation was performed when the soft tissue condition permitted (Wrinkle test positive). All procedures were performed by the same team. Patients with a tourniquet at the affect thigh were under anesthesia in the lateral position. An extensile lateral approach (ELA) or sinus tarsal approach (STA) was applied and then, with the flap was held by several 2.0-mm Kirschner wires, which allowed the visualization of the lateral wall of calcaneus and subtalar joint and anatomic reduction was performed directly under the guidance of C-arm fluoroscope. Briefly, after the subtalar joint reducted, and the height, length, width and deformity of calcaneus were corrected, and the pin was temporarily fixed till reach satisfactory adjustment of internal fixation by repeated intraoperative fluoroscope. The residual cavity in the calcaneus after resetting of the articular surface was filled and supported by implantation of allograft bone if necessary. Subsequently, plates and screws or solely cannulated screws were placed in the lateral wall of the calcaneus. The sustentaculum tail screws are inserted blindly from the lateral incision through the fracture block below the posterior talar articular surface, and the entry point is selected according to the fracture condition of the lateral wall and the location of the plate holes, 15°–30° forward and 10°–20° upward based on clinical experience. Thus, the screws can penetrate the medial cortex as far as possible, and the length should be strictly controlled to prevent irritation or injury to the medial vascular, nerve and tendon caused by excessive length of the screws.

### Postoperative management

All patients were prohibited from weight-bearing for 6–8 weeks after surgery, and partial weight-bearing was initial at 8–12 weeks, while full weight-bearing was allowed only after fracture healing examined by X-ray and CT scan. A total of 64 patients were selected for removal of plates and screws and other internal fixations 12–18 months after surgery.

### Clinical assessment

Pain intensity was quantified utilizing the visual analogue scale (VAS), where scores span from 0 to 10 points, representing a spectrum from absence of pain to the most severe pain imaginable. In our study, the visual analogue scale (VAS) scores, were meticulously collected both prior to the operation and at the final follow-up. Notably, the follow-up period for each patient extended to at least 1 years, ensuring a comprehensive evaluation over a significant duration. The functional outcomes were evaluated using the American Orthopaedic Foot and Ankle Society (AOFAS) ankle-hindfoot scores, which were recorded at pre-operation, post-operation, and at the final follow-up. This comprehensive score, ranging from 0 to 100 points, encompasses aspects of pain, functionality, and alignment. Additionally, radiographic assessments, including the measurement of Bohler’s angle and Gissane angle, were meticulously conducted on fluoroscopic images both before the operation and at the final follow-up period.

### Statistical

The Kolmogorov–Smirnov test was utilized to assess the normality of the variables. Differences between groups were evaluated using the Student's t-test for variables with a normal distribution, expressed as the mean ± standard deviation. For variables not normally distributed, the Mann–Whitney U test was used, presenting results as the median (interquartile range 25–75%). Categorical variables were analyzed with the chi-square test, reported as frequency (percentage). Longitudinal changes in Bohler's angle, Gissane's angle, and VAS scores between groups were compared using appropriate nonparametric tests. All statistical analyses were conducted via SPSS 20.0 software (SPSS, IL, USA), with a *P*-value of less than 0.05 denoting statistical significance.

## Results

### Patient information

A total of 73 feet from 72 patients were included in our study, with the ST group comprising 40 feet (29 male, 11 female) and the STF group comprising 33 feet (23 male, 9 female). The comparison of age, gender, side, fracture type, surgical access, fixation method and post-injury to surgery time between the two groups is shown in Table 1, and the differences were not statistically significant but comparable. The average age in ST group was 47.38 ± 13.49 while in STF group was 45.76 ± 14.74, and it showed no statistical differences (*P* = 0.626). Additionally, there showed no significant difference in age, gender, type of internal fixation, type of fractures. Patients in both groups were followed up for at least 12 months, with a mean of 13.37 months, and all cases reported no severe complications such as malunion or nonunion fractures and wound infections. No cases of vascular, nerve and tendon injury on the posterior aspect of the sustentaculum tail were found during the follow-up.

**Table 1:** Comparison of basic data of patients in ST group and STF group

	ST group	STF group	*P* value
Age, y	47.38 ± 13.49	45.76 ± 14.74	0.626
Gender, Male:Female	29:11	24:9	0.983
Sanders’ staging			0.261
Type II	20 (50.00%)	22 (66.67%)	
Type III	17 (42.50%)	8 (24.24%)	
Type IV	3 (7.50%)	3 (9.09%)	
Incision approach			0.283
ELA	17 (42.50%)	10 (30.30%)	
STA	23 (57.50%)	23 (69.70%)	
Fixation			0.115
Only screw	8 (20.00%)	14 (42.42%)	
Screw + microplate	15 (37.50%)	9 (27.28%)	
Screw + locking plate	17 (42.50%)	10 (30.30%)	

Data are presented as mean ± SD or percentage

### Functional outcome

All the functional outcomes including VAS scores, AOFAS scores, the Böhler’s angle, and the Gissane angle are presented in Table 2. The results of Böhler’s angle and the Gissane angle were significantly improved after surgery postoperatively and at final follow-up. In ST group, the Böhler's angle altered from 11.44 ± 5.94 to 31.39 ± 7.54 postoperatively, and changed to 30.61 ± 7.94 at final follow-up (*P* < 0.001) while the Gissane’s angle altered from 109.89 ± 12.13 to 121.23 ± 9.34 postoperatively, and changed to 119.08 ± 8.31 at final follow-up (*P* < 0.001). Similarly, in STF group, the Böhler's angle altered from 11.32 ± 6.77 to 28.82 ± 8.52 postoperatively, and changed to 28.25 ± 9.13 to 30.61 ± 7.94 at final follow-up (*P* < 0.001) while the Gissane’s angle altered from 110.47 ± 14.45 to 122.08 ± 8.84 postoperatively, and changed to 120.67 ± 9.07 at final follow-up (*P* < 0.001). However, there was no statistically significant difference between the two groups when comparing the calcaneus Böhler’s angle and Gissane’s angle preoperatively, 1 week postoperatively, and at 1 year follow-up (See Table 2).

**Table 2:** Comparison of Böhler’s angle and Gissane angle between ST group and STF group

	ST group	STF group	*P* value
Böhler’s angle (°)			
Preoperatively	11.44 ± 5.94	11.32 ± 6.77	0.936
1-week postoperatively	31.39 ± 7.54	28.82 ± 8.52	0.174
1-year postoperatively	30.61 ± 7.94	28.25 ± 9.13	0.241
Gissane angle (°)			
Preoperatively	109.89 ± 12.13	110.47 ± 14.45	0.854
1-week postoperatively	121.23 ± 9.34	122.08 ± 8.84	0.690
1-year postoperatively	119.08 ± 8.31	120.67 ± 9.07	0.437

Data are presented as mean ± SD

Results showed that VAS score was significantly different at postoperative comparing to preoperative in two groups (*P* < 0.001, *P* < 0.001, respectively). However, there was no significant difference in VAS scores before operation and at the final follow-up time between these two groups (*P* = 0.901, *P* = 0.180, respectively). AOFAS scores improved greatly after surgery, from 51.05 ± 10.70 to 88.95 ± 6.16 in the ST group and from 51.36 ± 10.66 to 89.78 ± 8.76 in the STF group, but there showed no significant difference between two groups at these time points (*P* = 0.0065, *P* = 0.757, respectively) (See Table 3).

**Table 3:** Intra-group comparison of VAS scores and AOFAS scores in two groups

	VAS scores		*P* value	AOFAS scores		*P* value
	Preoperative	Final follow-up		Preoperative	Final follow-up	
ST group	6.77 ± 1.07	0.83 ± 0.98	< 0.001	51.05 ± 10.70	88.95 ± 6.16	< 0.001
STF group	7.24 ± 1.12	1.03 ± 1.59	< 0.001	51.36 ± 10.66	89.78 ± 8.76	< 0.001

Data are presented as mean ± SD

## Discussion

Most calcaneal fractures impact the posterior articular surface, necessitating a reliable support point on the medial wall of the calcaneus for effective fracture fragment fixation, particularly in cases of osteoporotic or medial wall talus fractures. The talus, usually encased in tightly bound soft tissue, maintains an angle with the calcaneal body of less than 10 degrees or a displacement under 3 mm following most fractures. Given the rarity of simple talus fractures, it serves as a preferred anchor point in the lateral approach for calcaneal fracture management. This anchoring facilitates proper pressure distribution between bone blocks on the posterior articular surface, enabling anatomical repositioning and aiding in the restoration of the calcaneus's width. [12–14]

The sustentaculum tali, shaped roughly like a parallelogram, measures an average of 23.6 mm in length and width, and 9.5 mm in height. It forms a 27.7° upward convex angle and a 30.3° anterior inclination relative to the calcaneus's long axis. Featuring a cortical thickness of 2–3 mm, it possesses a finely dense inner trabecula running vertically. This dense zone is crucially linked to the trabecula beneath the calcaneus's posterior articular surface, playing a pivotal role in the calcaneus's structure. Most longitudinal pressure from the talus is transmitted to this area. Finite element analysis indicates that the anteromedial sustentaculum tali and the posterior calcaneus create a secondary stress zone in conjunction with the anterior middle base of the calcaneus [15]. The sustentaculum tali's limited size, irregular shape, and the merged articular surfaces beneath the calcaneus talus complicate precise intraoperative screw placement, presenting a steep learning curve. Currently, surgeons rely on experience for sustentaculum tali screw placement, often navigating blindly from the calcaneal bone's lateral wall through the fracture fragment below the posterior articular surface. This process typically necessitates multiple attempts and frequent fluoroscopic checks to ensure effective fixation during surgery [16], 17 Therefore, it is likely to result in increased intraoperative fluoroscope, prolonged operative time, and risk of injury to the medial vascular nerve bundle [18]. Moreover, the location of the screw hole of the lateral calcaneus plate also restricts the screw orientation to a large angular tilt, and the accurate placement rate of the sustentaculum tail screws cannot reach satisfactory. In recent years, some scholars have proposed the use of arthroscopic targeting devices or preoperative prefabricated templates to improve the accuracy of screw placement into the sustentaculum tail, but the actual clinical situation is limited by many factors and difficult to promote [19–24]. Theoretically, the precise placement point and direction of sustentaculum tali screws in calcaneal fractures, crucial for clinical guidance, are still subjects of debate. Our study focused on exact placement of these screws, categorizing cases as sustentaculum fragment fixation when screws passed through the first continuous bone trabecula lateral to the calcaneus's central triangle but outside the conventional sustentaculum tali range. We compared the effectiveness of this approach against displaced intra-articular calcaneal fractures to refine screw placement strategies for clinical practice [11], 25–27.

Surgical treatment of calcaneal fractures helps to reposition the articular surface and achieve reliable compression between the fracture blocks through internal fixation, and internal fixation with sustentaculum tail screws helps to maintain the stability of the re-positioned posterior talar articular surface [28–30], forming a framework structure to restore the calcaneus shape until bone healing [31–33]. Significantly, there were notable differences in Gissane's and Böhler's angles before surgery and one week post-surgery (*P* < 0.05), but these angles remained stable without loss of repositioning up to one year post-surgery. Also, one year post-surgery, the differences in American Orthopaedic Foot and Ankle Society (AOFAS) scores and visual analogue scale (VAS) scores between the groups were not statistically significant. This indicates that precise screw placement in the sustentaculum tali does not significantly affect short-term foot function restoration or pain reduction, affirming similar clinical effectiveness of sustentaculum tali and sustentaculum tali fragment approaches. Furthermore, a computer-simulated biomechanical study revealed that the placement of trans-plated screws, whether in the sustentaculum tali or not, does not impact the overall force and displacement on the posterior joint surface when a locking plate is used [34, 35]. Additionally, according to the result of study, although the screws were not fixed to the sustentaculum tail in the experiment, but at least one screw was fixed to the sustentaculum fragment, resulting in similar clinical efficacy, which also provided mechanical corroboration evidence to this study.

The importance of accurately placing sustentaculum tali screws in calcaneal fracture surgery is well-recognized, yet achieving this precision is challenging. Our review assessed calcaneal reconstruction and functional recovery in groups with both precise and imprecise screw placement, revealing that suboptimal placement did not significantly impact short-term surgical outcomes. This suggests that the sustentaculum tali is not an isolated fixation target and allows for a broader range of fixation. We introduce the concept of 'sustentaculum tali fragment', defined by a specific bone trabecula. Even when screws did not precisely target the sustentaculum tali, effective fixation was achieved in the sustentaculum fragment, providing anchorage, lateral compression, and reducing postoperative displacement. Fixation to the sustentaculum fragment, being more extensive and feasible in clinical practice, helps shorten operation time, reduce induced injuries and complications, while delivering similar therapeutic outcomes. Therefore, we recommend fixing screws either to the sustentaculum tali for optimal support and anchorage or directly to the sustentaculum fragment to minimize injury in challenging procedures. This approach aims to improve functional outcome and reduce the duration of hospitalization, which are consistent with previous published results [36]. Extended hospital stays and complications delay rehabilitation, and the time away from work and recreational activities can be significantly prolonged when a second surgery becomes necessary.

However, when patients suffering from debilitating pain or severe deformity of the hindfoot, tibiotalocalcaneal (TTC) arthrodesis serves as an effective treatment option, which not only alleviates pain but also corrects malalignment, serving as a vital alternative to amputation, supported by both clinical practice and existing literature [37–39]. Therefore, the possibility of progressing to TTC arthrodesis should be an integral part of the treatment strategy for calcaneal fractures, especially in cases with poor initial outcomes or when severe complications arise [40].

## Limitations

This study has its own limitations. Firstly, the main limitation of research is that it is a retrospective analysis and randomized clinical trials (RCTs) are insufficient. Therefore, there are inherent shortcomings presenting in our research. Moreover, patients’ data and information are collected from a single center, lacking data from multi-center, which resulting in single center analysis bias. Thirdly, the overall follow-up period of cases was limited to one year only, lacking the long-term follow up efficacy in ST and STF group. Thirdly, only patients classified as Sanders type II/IV were enrolled in our research, which is hardly representative of all types of calcaneal fractures, and therefore future RCTs combined with other types of calcaneus fractures such as Sanders I, malunion, and nonunion should be taken into consideration. While the study does not raise any ethical concerns, its limited scope, conducted at a single center, may not capture the diverse range of clinical scenarios and patient outcomes. This single-center approach restricts the generalizability of the findings, as it may not adequately represent the variability in clinical practices and patient demographics encountered in a wider, multi-center context.

## Conclusion

In Summary, our result presented that sustentaculum tali screw fixation to the medial wall of calcaneus, also term as sustentaculum tali fragment, provided reliable anchorage, restoring the overall continuity of the calcaneus and achieving the goal of surgical treatment.
